# Use of the superficial inferior epigastric vein in breast reconstruction with a deep inferior epigastric artery perforator flap

**DOI:** 10.3389/fsurg.2023.1050172

**Published:** 2023-05-22

**Authors:** Charalampos Varnava, Philipp Wiebringhaus, David Kampshoff, Tobias Hirsch, Maximilian Kueckelhaus

**Affiliations:** ^1^ Division of Plastic and Reconstructive Surgery, Department of Trauma, Hand and Reconstructive Surgery, University Hospital Muenster, Muenster, Germany; ^2^ Department of Plastic, Reconstructive and Aesthetic Surgery, Hand Surgery, Fachklinik Hornheide, Muenster, Germany; ^3^ Department of Plastic and Reconstructive Surgery, Institute of Musculoskeletal Medicine, University of Muenster, Muenster, Germany

**Keywords:** DIEP, SIEV, perforator, breast reconstruction, microsurgery, coupler

## Abstract

**Background:**

Autologous breast reconstruction is highly regarded in reconstructive surgery after mastectomy. DIEP flap reconstruction represents the gold standard for autologous breast reconstruction. The major advantages of DIEP flap reconstruction are its adequate volume, large vascular caliber and pedicle length. Despite reliable anatomy, there are procedures where the plastic surgeon's creativity is required, not only to shape the new breast, but also to overcome microsurgical challenges. An important tool in these cases is the superficial epigastric vein (SIEV).

**Methods:**

150 DIEP flap procedures performed between 2018 and 2021 were retrospectively evaluated for SIEV use. Intraoperative and postoperative data were analyzed. Rate of anastomosis revision, total and partial flap loss, fat necrosis and donor site complications were evaluated.

**Results:**

In a total of 150 breast reconstructions with a DIEP flap performed in our clinic, the SIEV was used in 5 cases. The indication for using the SIEV was to improve the venous drainage of the flap or as a graft to reconstruct the main artery perforator. Among the 5 cases, no flap loss occurred.

**Conclusions:**

Use of the SIEV is an excellent method to expand the microsurgical options in breast reconstruction with DIEP flap surgery. It provides a safe and reliable procedure to improve venous outflow in cases of inadequate outflow from the deep venous system. The SIEV could also provide a very good option for fast and reliable application as an interposition device in case of arterial complications.

## Introduction

Breast reconstruction is one of the main fields of interest and practice of many plastic surgeons. Over the years, many different options adapted to the needs of the individual patient were developed. The gold standard for autologous breast reconstruction is the deep inferior epigastric artery perforator (DIEP) flap, because of its high volume, the reliable anatomy and length of the perforators and the convenient intraoperative positioning of the patient (1). The flap can be harvested in supine position while there is no need for repositioning of the patient during surgery, reducing duration of surgery.

Although the flap anatomy is reliable, there are cases where there is a need for improvisation and use of the microsurgeon's creativity. To achieve a harmonic breast form and to successfully perform the microsurgical part of the surgery can possess a challenge even for an experienced reconstructive breast surgeon.

The superficial inferior epigastric vein (SIEV) can be a powerful tool in cases where the route of the surgery deviates from the norm. It can be utilized to improve flap drainage and as a vessel graft. In this study, we report our results of SIEV application in DIEP flap breast reconstruction.

## Materials and methods

In this study, we analyzed the first 150 DIEP flaps that we performed between March 2018 and May 2021. Clinical data were procured from the hospital's free flap database and patient records. Rate of anastomosis revision, total and partial flap loss, fat necrosis and donor site complications (seroma, infection) were evaluated. We performed a literature review and we discuss numerous aspects regarding the SIEV and its use in breast reconstruction with the DIEP flap.

### Preoperative procedures

Each patient preoperatively underwent a computer tomography angiography (CTA) for mapping of the deep inferior epigastric artery and its perforators. One day before surgery, perforators were identified with a hand-held doppler ultrasound device. No special attention was given to flap veins or specifically the SIEV.

### Flap harvest and SIEV

DIEP flap elevation was performed in a supine position. The procedures were conducted in a two-team approach. During abdominal dissection the SIEV was preserved at a length of 6 cm. The superficial inferior epigastric artery and its accompanying vein (vena comitans) were not routinely preserved.

**Figure 1:**
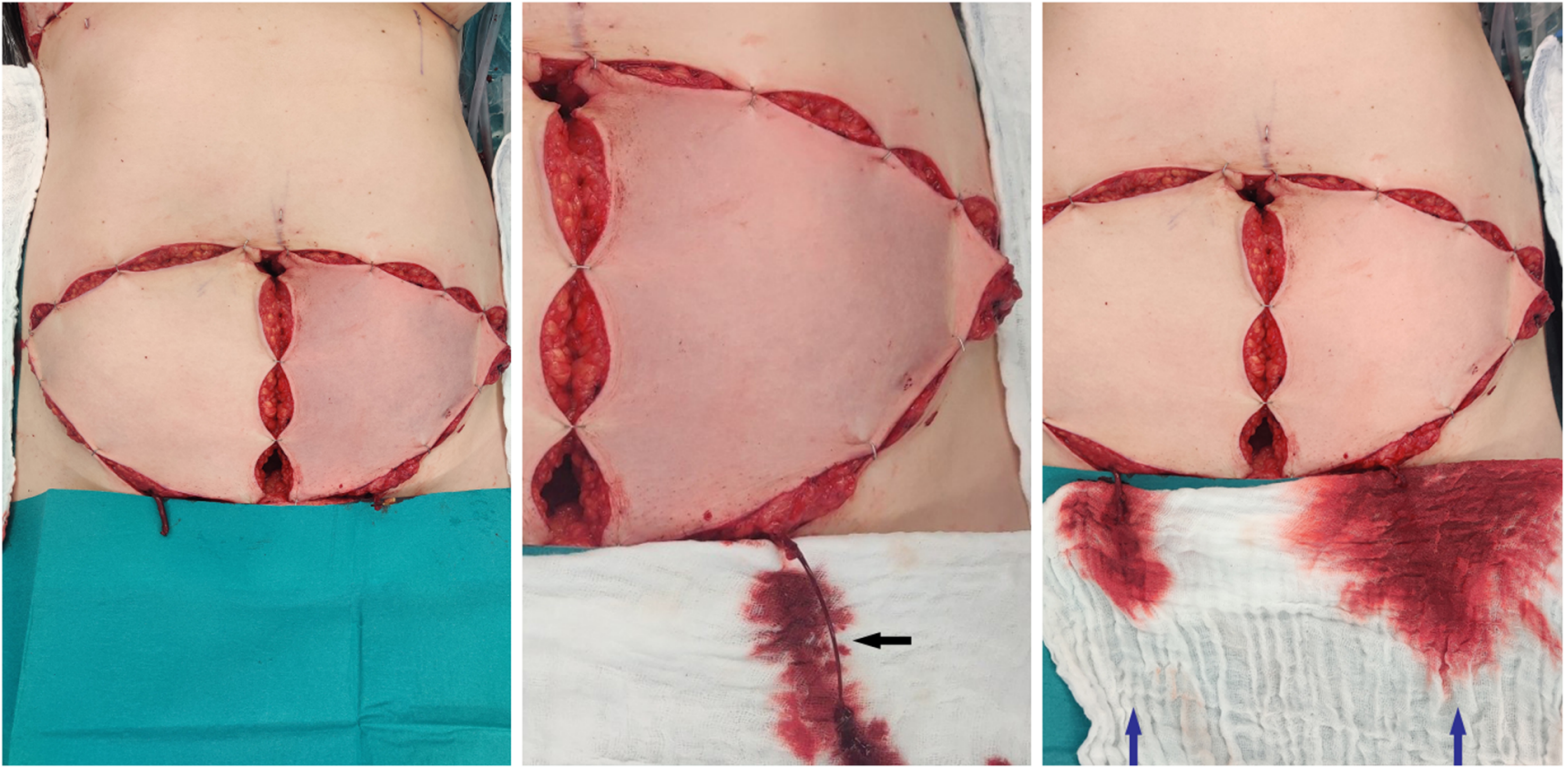
Left picture: both flaps isolated on the perforators with clamped SIEV, the left flap shows a venous congestion, the right flap shows a normal capillary refill time in spite of a congested SIEV. Middle picture: After releasing the clamp of the SIEV presentation of an increased pressure on the superficial venous system (black arrow) Right picture: After releasing the clamp of the SIEV on both clamps recovery of the left flap. Increased venous flow through the SIEV on the left flap compared to the right flap (blue arrows).

Following perforator-based flap elevation, the flap was inspected and color and capillary refill time were evaluated. In case of a venous congestion (identified as a purplish skin color with brisk capillary refill) the SIEV was released. (Figure 1) If the venous flow consequentially improved (normal skin color and capillary refill), the decision was made to superdrain the flap and connect the SIEV. If the flap remained congested despite the release of the SIEV then the flap was not used at all.

### Anastomoses

The venous anastomoses were always performed using coupler rings (Synovis Micro Companies Alliance, Inc., Alabama, USA).

### Data collection

Peri- and intraoperative measures and clinical outcomes were analyzed using our electronic patient records.

### Statistics

The retrieved data was documented in Excel-Tables. The statistical analysis was made with Microsoft Excel v2104 (Microsoft, Redmond, WA, USA) and IBM SPSS Statistics v25 (IBM, Armonk, NY, USA).

We tested for normality using the Kolmogorov-Smirnov test. The equality of variances was assessed using the Lavene test. The variables of each group were compared using the Fischeŕs exact test and the Mann-Whitney test.

A *p*-value of < 0.05 was considered to be statistically significant.

## Results

In a total of 150 breast reconstructions with a DIEP flap performed in our clinic, the SIEV was used in five cases. In four cases (2.7%) the SIEV was used to improve the venous flow of the flap (Table 1). In four cases it was used as a vein graft. In three cases the SIEV was used both as a vein graft and for the improvement of the venous flow of the flap. Two of four patients who needed a SIEV were taken back to the operating theater due to a venous congestion postoperatively. No other statistically significant differences were found between the “no SIEV” and the “SIEV” groups with reference to the complication rates (Table 2). In one case the SIEV was used to reconstruct an injured perforator. Among the 5 cases no flap loss occurred.

**Table 1:** Patient characteristics (SIEV use due to venous congestion) Mean ± SD.

	no SIEV (%)	SIEV (%)	*p*
No. of flaps			
Total	146/150 (97.3)	4/150 (2.7)	
Unilateral	62/64 (96.9)	2/64 (3.1)	
Bilateral	84/86 (97.7)	2/86 (2.3)	
Age (years)			
	50.3 ± 10.4	55.8 ± 6.4	0.286
BMI (Kg/m^2^)			
	27.5 ± 4.7	30.5 ± 5.0	0.208
Comorbidities			
Nicotine	16/103 (15.5)	0/4 (0.0)	1.000
Diabetes mellitus	2/103 (1.9)	0/4 (0.0)	1.000
Arterial hypertension	28/103 (27.2)	1/4 (25.0)	1.000
Breast			
BRCA	24/103 (23.3)	1/4 (25.0)	1.000
Mamma-Ca	83/103 (80.6)	4/4 (100.0)	1.000

SD, Standard Deviation.

**Table 2:** Complications (SIEV use due to venous congestion).

	no SIEV (%)	SIEV (%)	*P*
Total flap loss	4/146 (2.7)	0/4 (0.0)	1.000
Partial flap loss	6/146 (4.1)	0/4 (0.0)	1.000
Fat necrosis	20/146 (13.7)	1/4 (25.0)	1.000
Anastomosis revision	7/146 (4.8)	2/4 (50.0)	0.018^*^
Infection donor site	8/103 (7.8)	0/4 (0.0)	1.000
Seroma donor site	14/103 (13.6)	0/4 (0.0)	0.457

^*^ Statistically significant.

### Venous congestion

**Figure 2:**
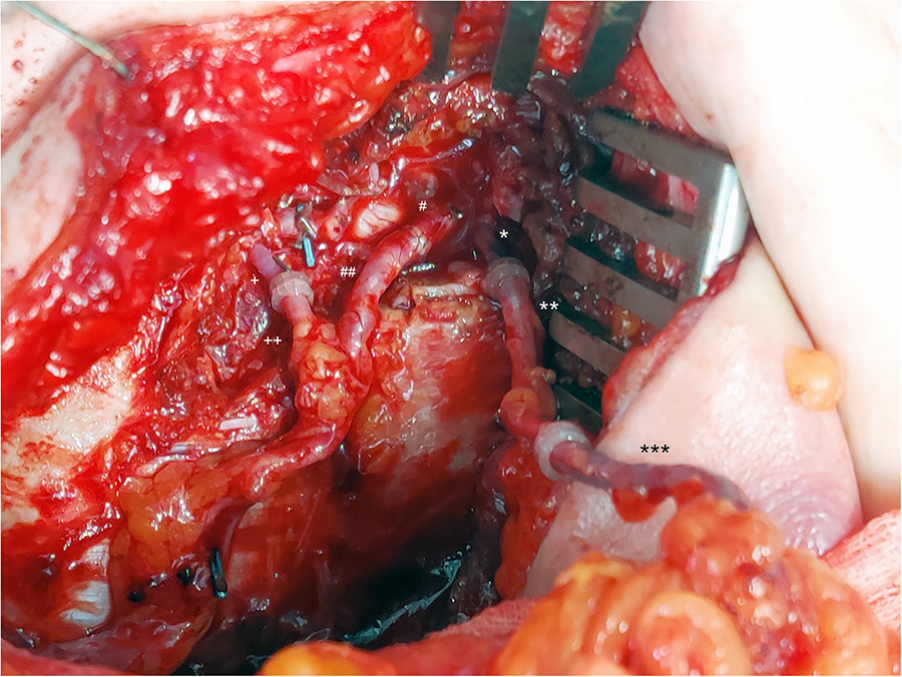
SIEV as a vein graft and for improvement of the venous flow of a DIEP flap. *: IMV cranial, **: contralateral SIEV (vein graft), ***: ipsilateral SIEV. #: IMA cranial, ##: DIEA. +: IMV caudal, ++: DIEV.

In four cases the use of the ipsilateral SIEV was needed to improve the venous flow of a congested flap. One of those times the SIEV length was enough to perform the anastomosis and in the remaining three a vein graft was needed. In all three cases the contralateral SIEV was used as a graft (Figure 2). Two out of four cases needed to be taken back to the operating theater due to venous thrombosis. All four flaps survived.

### Injured perforator

Our final case was that of a 37-year-old patient who presented to our clinic to undergo a bilateral prophylactic mastectomy and breast reconstruction due to a BRCA-1 mutation.

**Figure 3:**
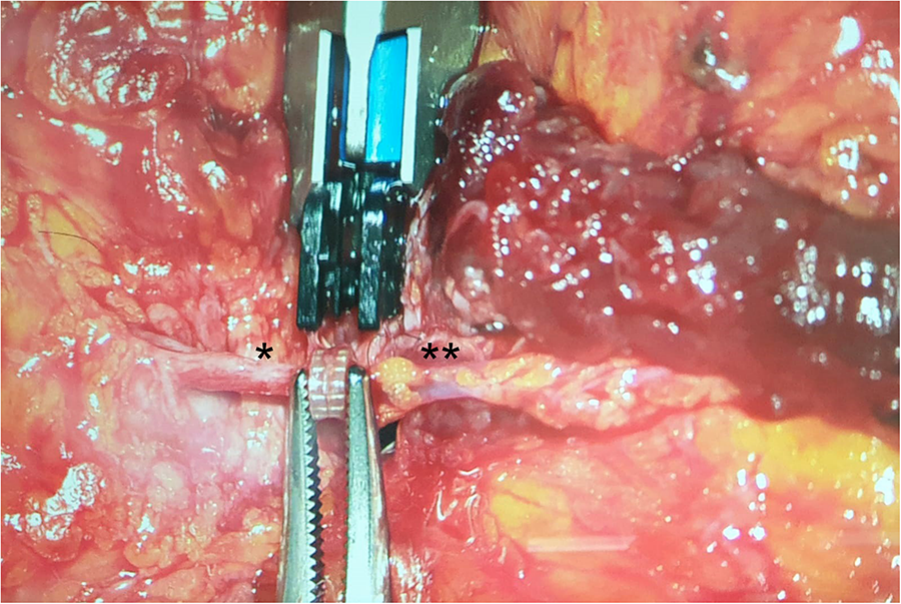
Coupler-Anastomosis between the cranial segment of the perforator and the SIEV. *: perforator, **: SIEV.

**Figure 4:**
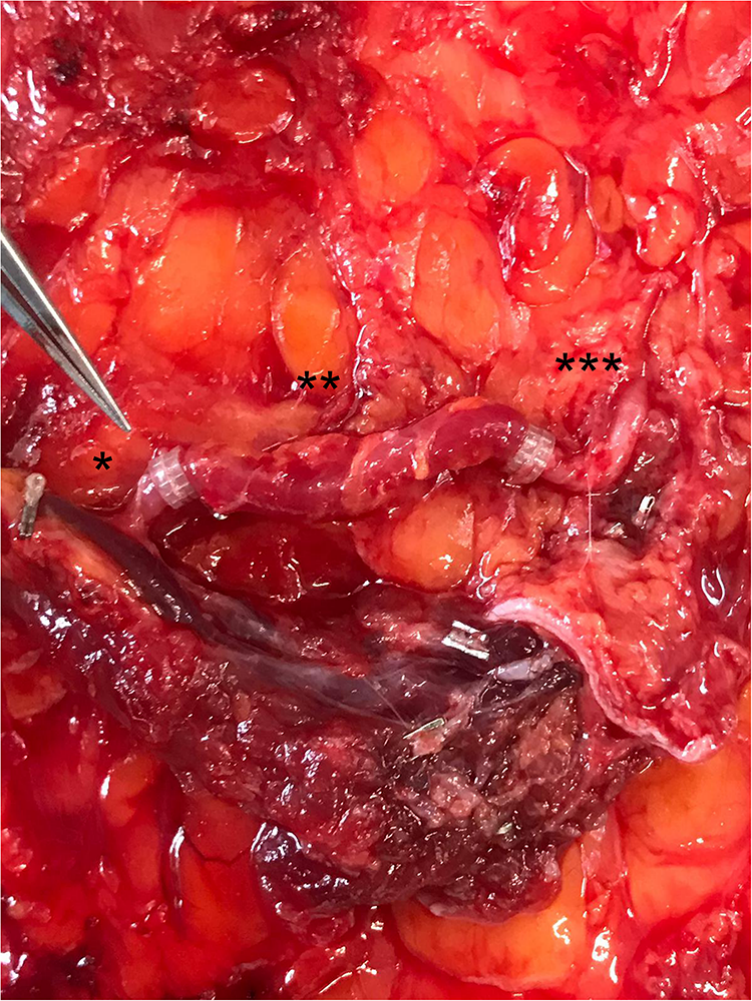
Completed perforator-reconstruction with the SIEV (after releasing the clamps and restoring the arterial flow). *: caudal perforator segment, **: SIEV, ***: cranial perforator segment.

**Figure 5:**
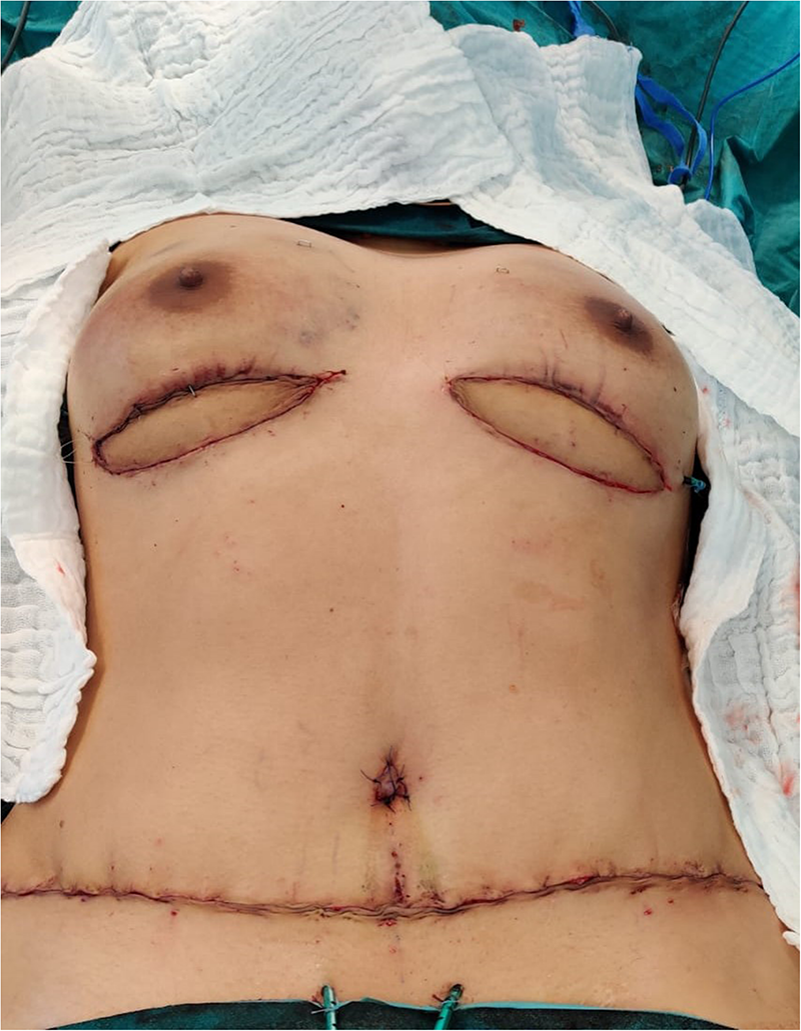
Postoperative on-table photograph. The SIEV was used on the flap of the left breast.

The procedure was performed by four surgeons. During the harvesting of the second DIEP flap from the left side of the abdomen it sustained an injury to the arterial segment of the only sufficiently sized perforator. The injury was not perceived at the time it happened. After the completion of the pedicle dissection the flap showed no capillary refill. Inspecting the flap, perforator, and pedicle we found a coagulation injury on the arterial segment of the perforator. Excising the coagulated vessel, a primary suture of the vessel ends was not possible due to the length of the artery gap, which necessitated the use of a graft. We decided to use the contralateral SIEV as a reversed vein graft, which was already dissected initally at a length of 7 cm, to reconstruct the injured arterial segment of the perforator (Figures 3, 4, Supplementary Video S1) We performed the arterio-venous anastomoses using 2 × 1.5 mm coupler rings (Synovis Micro Companies Alliance, Inc., Alabama, USA). After releasing the clamps, the flap skin showed a pink color with a normal capillary refill. Simultaneously, the right breast was reconstructed with the DIEP flap harvested from the right side of the abdomen. The left flap was then connected with an end-to-end arterial anastomosis to the internal mammary artery (sutured) and a venous anastomosis to the internal mammary vein. Upon completion of the anastomoses, the flap showed a normal capillary refill time and the perforator showed a good doppler signal (Figure 5).

**Figure 6:**
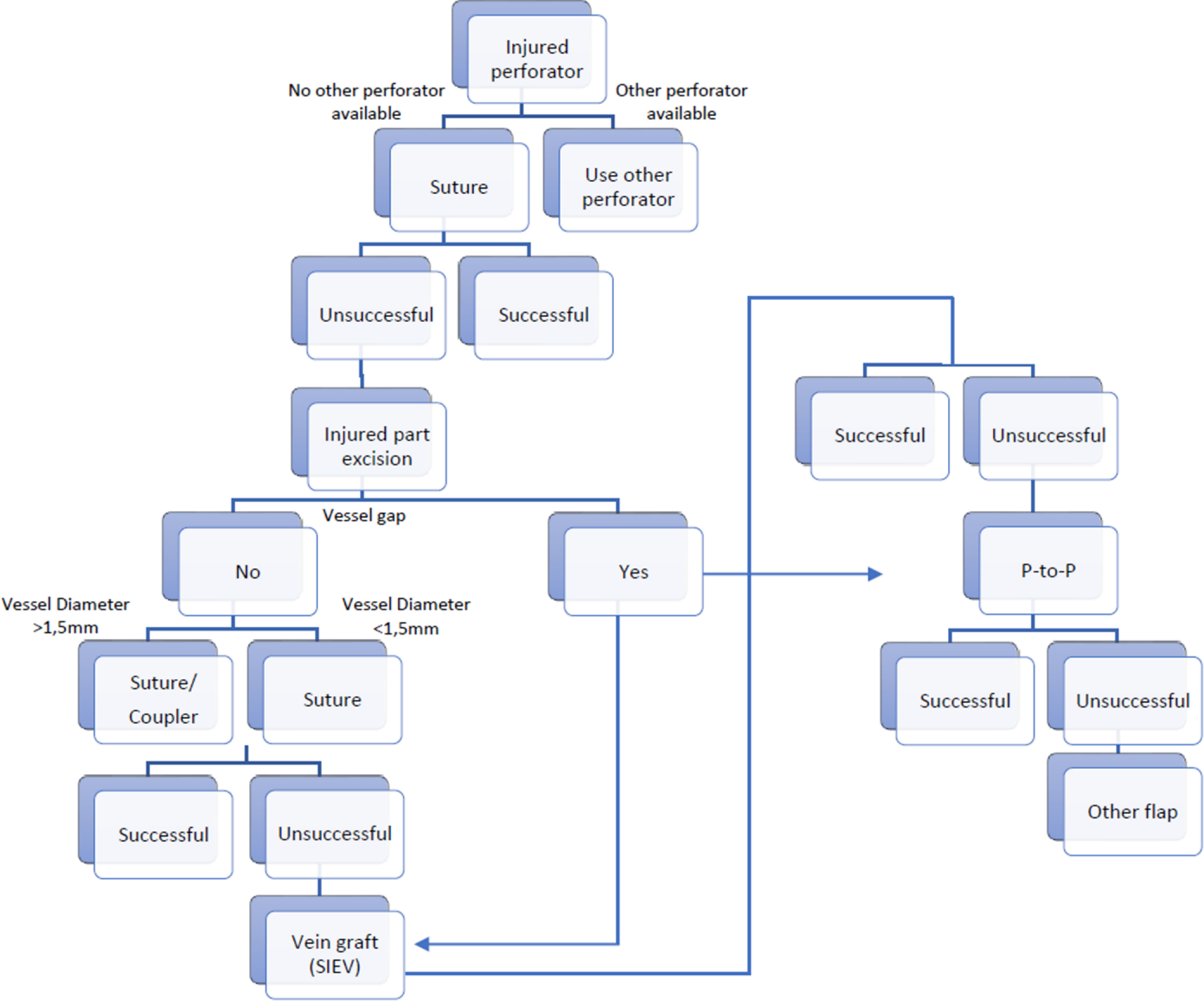
Decisional tree on how to proceed in a case of an injured perforator and when the use of the SIEV can be considered. P-to-P: Perforator to perforator anastomosis.

However, the right flap then exhibited a disturbance in the perfusion with no capillary refill. A revision of the anastomosis revealed a thrombus in the arterial anastomosis. We performed a thrombectomy and successfully reconnected the vessels. At that point the patient received an intravenous injection of 500 mg of Aspirin, which is routinely used in our institution in cases of early diagnosed arterial thrombosis. After completing the revision of the right side, we observed an anomaly in the perfusion with no capillary refill on the left side. Inspecting the pedicle showed a thrombus on the distal end of the vein graft. The coupler ring and the thrombus were then removed, and the SIEV was again connected to the perforator with a 1.5 mm coupler ring. The flap again showed a pink color with normal capillary refill time and doppler signal. There were no other incidents during the operation. The duration of the surgery was 556 min.

## Discussion

Autologous breast reconstruction using a DIEP flap is a complex procedure with many steps that can potentially go wrong. Due to the high patient satisfaction (2) and the acceptable complication rate despite its complexity, this procedure is constantly gaining supporters. Intraoperative complications include perforator injury, venous congestion, and microvascular complications (3). It is crucial for the reconstructive surgeon to be aware of the potential pitfalls and to have the knowledge and creativity to cope with each complication. SIEV usage in salvage procedures was addressed previously in the literature (4–19). Most commonly it is used to improve the venous flow of a congested flap or as a vein graft taken from the contralateral side to lengthen the SIEV ipsilaterally. The use of a secondary vein in the drainage of a DIEP flap is reported to significantly reduce the incidence of venous congestion (20). Using the SIEV as a routine method to superdrain the diep flap is also described in the literature (13, 14, 21). However, this technique does not seem to be adapted by the majority.

Karadsheh et al. provided a theoretical model describing the dynamics of venous flow in the DIEP flap (22).

Numerous cadaveric and clinical studies provided anatomical details on the venous drainage of the lower abdomen (23–48). Gusenoff et al. reported on the correlation between body mass index (BMI) and pannus weight, and presence and size of the superficial inferior epigastric vessels (49). Bast et al. demonstrated also that patients with suprascarpal fat pad thicker as 23 mm had larger SIEVs irrespective of the deep system perforators (50). Huang et al. also demonstrated that suprascarpal fat pad thickness was positively correlated with SIEV diameter. They suggested that the risk of venous congestion is significantly increased with thinner surprascarpal fat pads and recommended prophylactic SIEV dissection in all patients with suprascarpal fat pad thickness less than 18 mm (51). Ayhan et al. reported a slightly inverse correlation between the size of SIEV and deep inferior epigastric vein (DIEV), although this was not statistically significant (52). Figus et al. demonstrated also a non-significant inverse correlation with the DIEV. Interestingly, they found no significant difference in the diameters of the deep inferior epigastric artery (DIEA), DIEV, perforating arteries or perforating veins when comparing patients with and without an identifiable SIEV (53). Smit et al. reported an increase in the venous pressure of the SIEV after raising a DIEP flap on a single perforator compared to the pressure at the beginning of the dissection. In one of the 26 cases clinical signs of venous congestion were observed. In this case, the increase in venous pressure was the highest (54). Lie et al. demonstrated the presence of two main types of venous communications between the superficial and deep venous systems: the large-caliber venae communicantes and small-caliber venae commitantes (55). Schaverien et al. suggested after comparing breast reconstruction outcomes with a DIEP flap, with preoperative radiologic reporting of contrast-enhanced magnetic resonance angiographic imaging, that the selection of perforators with direct venous connections with the SIEV of suitable caliber is likely to significantly reduce the risk of venous complications (56). Frank et al. demonstrated that the larger the diameter of a perforator, the more likely the perforator had a connection to the SIEV or the superficial fat pad. Additionally, medial row perforators showed more direct connections to the SIEV compared to lateral row perforators (57). Rothenberger et al. suggested that the supercharging of the contralateral SIEV leads to an improved venous outflow particularly in flaps containing larger proportions of the contralateral zones (58). Also in patients with the need of a large flap it was possible to use a unipedicle four-zone DIEP flap with an additional anastomosis of the SIEV, if the superficial venous system showed strong vascular connections between right and left hemiabdomen (59).

Recipient vessel for the SIEV can be any vein with an adequate caliber and length. Described in the literature are, among others, the thoracoacromial vein (60, 61), the lateral thoracic vein (12, 62, 63), the circumflex scapular vein (5, 64), the thoracodorsal vein, the basilic (65) und cephalic vein (6, 17, 66, 67), the external jugular vein (9, 59), the internal mammary vein (7, 11, 68–70) and its perforators (71). Rohde et al. and later Sbitany et al. described a flap salvage technique by anastomosing the ipsilateral SIEV to a venae comitantes of the deep inferior epigastric pedicle (72, 73). Liu et al. described a flap salvage technique by anastomosing the ipsilateral SIEV to the unused DIEV pedicle in a reverse venous flow fashion (74). Xin et al. described the reverse flow anastomosis of the contralateral SIEV to the ipsilateral SIEV as an efficient method of venous augmentation for the DIEP flap (75). Teven et al. reported after removal of a Port-a-cath the anastomosis of the SIEV to the fibrous capsular sheath that had formed around the catheter (76). Pignatti et al. developed an algorithm to help the surgeon in the choice of the veins for the superdrainage anastomosis (77).

In cases where the SIEV is not long enough or the flap positioning is not convenient for the direct connection to a recipient vein, a vein graft can be used to lengthen the vessel. Appropriate veins to be used as a graft are also all veins with a sufficient caliber and length, for example the great saphenous vein (5), the cephalic vein, the DIEV (7), the superficial circumflex iliac vein (SCIV) (14) and the SIEV (71, 78). The DIEA is also reported to be used as a graft between the SIEV and the internal mammary vein (IMV) (11).

The use of a cannula to intermittent superdischarge the flap through the SIEV (9, 10, 79) as well as the use of SIEV alone in a setting of absent drainage *via* the DIEV (7) or by given evidence of sufficient venous outflow (80) are also described in the literature. The use of a “superficial vein-only” DIEP flap in cases of venous congestion and when the perforator veins were deemed much smaller than the SIEV was also described recently (81).

Yoshimatsu et al. applied intraoperative indocyanine green (ICG) angiography for detecting flap congestion. They showed a difference in the ICG images of congested flaps before and after releasing the SIEV and suggested that ICG angiography can detect not only ischemia but also congestion of a flap (82).

In this study we discuss our experience after 150 breast reconstructions with a DIEP flap and we demonstrate first time in literature the use of the contralateral SIEV to restore the continuity of an injured arterial segment of a DIEP perforator performing an anastomosis with a microvascular coupler device.

There are reconstruction centers which routinely use composite interposition grafts when performing specific flaps like the lumbar artery perforator flap in breast reconstruction (83).

Both for venous anastomoses as well as for the arterial anastomoses there is data supporting that the use of coupler devices is a safe and efficient alternative to hand-sewn anastomosis (84, 85).

A metaanalysis published in 2015 revealed that superdrainage with SIEV significantly lowers the probability of flap congestion while having minimal effect on flap survival. In terms of partial flap necrosis general trends toward lower risks were identified, without being statistically significant (86). Another metaanalysis published in 2021 showed a statistically significant advantage of super-drainage to reduce venous congestion of the flap, partial flap necrosis, total flap necrosis and the need to take the patient back to surgery for perfusion-related complications (87). A systematic review of the literature published in 2015 reported an incidence of intraoperative venous congestion of the free abdominal flap during breast reconstruction of 2.8%. This was attributed to the persistent dominance of the superficial venous system and disconnection between the superficial and deep venous system (88). In our study, four out of a hundred and fifty flaps (2.7%) necessitated the use of the SIEV to improve the venous flow of the flap. Two out of four superdrained flaps needed to be taken back to the operating theater. However, the fact that these two revisions due to venous congestion took place at the beginning of our learning curve could possibly also have an impact on this result.

Disadvantages of always preserving the SIEV may be prolonged operating times and the possibility of increased donor site complications, such as seroma.

The time needed for harvesting the SIEV is acceptable (21). In our experience, no longer than ten minutes are needed for the dissection of the SIEV at a minimum length of six centimeters. Bartlett et al. reported also no statistically significant difference in operative time between a group of patients who required additional venous anastomoses due to intraoperative venous congestion and a control group (89). However, in a study of 404 patients the harvest of the SIEV and the number of venous anastomoses were negatively associated with the total surgery time (90). A study of 100 consecutive cases of DIEP flap reconstruction reported that bilateral SIEV dissection increased seroma rates significantly compared to the control group (without SIEV dissection). Interestingly, unilateral SIEV dissection did not increase seroma rates or length of hospital stay significantly (91).

Blondeel et al. reported to have noticed, although not documented, that all cases in which a venous congestion within the superficial venous system occurred were associated with larger than usual superficial inferior epigastric veins (4). That corresponds with our clinical experience as well. However, a study of 39 DIEP flaps showed no correlation between the radiographic size or the *in situ* size of the SIEV, the BMI, the age or the BMI:SIEV size ratio and the need for SIEV use (92).

Salvage procedures after accidental disruption of a perforator were already described in some reconstructive fields including breast reconstruction (93). In most cases, a direct repair/anastomosis was performed. In one case an anterolateral thigh (ALT) flap was used for the coverage of a chronic ulcer of patient with a methicillin resistant Staphylococcus aureus (MRSA) infection. A vein graft was necessary for elongation of the arterial perforator segment. The flap did not survive. According to the authors, even though the patient was administered antibiotics, the small perforator repaired with a vein graft possibly was not able to withstand invasion by the remaining toxic bacteria (94). In a second case where a vein graft was used the fibula flap survived but sustained marginal necrosis (93). The decision-making process for managing an injured perforator may be illustrated using a decision tree, such as the example shown in Figure 6.

In our case, the DIEP flap survived after the repair of the arterial perforator segment with the contralateral SIEV and demonstrated only a small area of fat necrosis.

Despite the evolution of microsurgery, supermicrosurgery and the technical refinements proposed by experienced microsurgeons (95–100), there will probably always be cases in which an unexpected error and an inadvertent injury of the perforator vessels will occur. Our solution to reconstruct the perforator with a SIEV graft using a microsurgical coupler device adds to the diverse solutions made possible by the SIEV and gives weight to the idea of preserving the SIEV while harvesting the DIEP flap.

This study had the limitations of being retrospective in design and having a small sample size especially in the SIEV group.

Although a *p*-value of < 0.05 was considered to be statistically significant, the small number of SIEV cases led to a low statistical power.

## Conclusion

The SIEV is an effective way to broaden the microsurgical choices in breast reconstruction using DIEP flap surgery. It provides a safe and reliable option to improve venous outflow in cases of inadequate outflow from the deep venous system and it may potentially be a good alternative for fast and reliable application as an interposition graft in case of perforator injuries.

Although only 2.7% of our DIEP flaps needed drainage of the SIEV, we routinely preserve the SIEV for the previously mentioned reasons. Especially if bilateral breast reconstruction is performed, attention to preserving the SIEV should be paid.

## Data Availability

The raw data supporting the conclusions of this article will be made available by the authors, without undue reservation.

## Supplementary material

SUPPLEMENTARY VIDEO 1

Pulsating perforator after Perforator-Reconstruction with SIEV.
